# Extra-Axial Skeletal Metastasis of Malignant Melanoma: Case Report and Literature Review

**DOI:** 10.7759/cureus.22115

**Published:** 2022-02-11

**Authors:** Keerthi Gullapalli, Priyal Agarwal, Osama Mosalem, Venumadhavi Gogineni, Richa Tikaria

**Affiliations:** 1 Internal Medicine, Michigan State University-Sparrow Hospital, Lansing, USA

**Keywords:** immunotherapy, denosumab, trametinib, dabrafenib, extra-axial metastasis, appendicular skeletal metastasis, skeletal related events, pathological fracture, braf mutation, malignant melanoma

## Abstract

The incidence of malignant melanoma is increasing worldwide and is one of the major causes of skin cancer deaths in the United States. Although melanoma has the potential to metastasize to any organ, the incidence of bone metastasis is low (~25%) compared to liver or lung metastasis. However, when a bone is involved, metastasis occurs to the axial skeleton in most cases (80%-90%), and involvement of the appendicular skeleton is relatively rare. We here describe the case of a patient who presented with a pathological fracture due to extra-axial skeletal metastasis of a widespread malignant melanoma.

A 45-year-old female with an unremarkable past medical history presented to the ED with acute left hip pain. X-ray demonstrated left intertrochanteric femur fracture with an abnormal, suspicious lesion at the fracture site. Detailed physical examination revealed various skin nodules on the anterior chest wall, right upper back, and left cheek. CT of the chest/abdomen/pelvis (CT C/A/P) showed multiple lytic bone lesions and metastatic lesions in lungs, soft tissue, and mediastinal lymph nodes. She underwent surgical stabilization of the fracture, and a biopsy of the bone lesion revealed metastatic malignant melanoma with BRAF V600E mutation. She was started on localized radiotherapy followed by targeted therapy (dabrafenib and trametinib) and denosumab for her stage IV (cTX, cN2, cM1b(1)) (American Joint Committee on Cancer [AJCC] cancer staging 8th edition) disease. Despite treatment, her disease progressed as evidenced by the presence of new metastatic foci on a positron emission tomography-computed tomography (PET-CT) scan performed at a three-month follow-up. Her clinical course was complicated by hemoperitoneum due to bleeding from metastatic liver lesions and respiratory failure requiring a prolonged stay in the ICU before she was deceased.

In most cases, malignant melanoma presents with skin lesions at an early stage. Very few patients (4%) have metastatic disease at presentation. Although metastasis to bone is known to occur in advanced disease, involvement of the extra-axial skeleton is relatively rare. Malignant melanoma, initially presenting as pathological fracture of the appendicular skeleton, is not commonly encountered. Our case emphasizes the aggressive nature of malignant melanoma with an aim to raise physicians' awareness of this uncommon presentation. A brief review of the literature exploring prognosis and currently available treatment options is discussed.

## Introduction

Malignant melanoma is one of the most aggressive forms of skin cancer in the United States [1]. Its incidence is increasing worldwide, and while it still represents less than 5% of all cutaneous malignancies, it accounts for approximately 50%-70% of deaths from skin cancer [1]. Melanoma affects a higher proportion of younger patients and there is a female preponderance in the age group (4:10 in 20-24-year-old) which changes to a male preponderance (16:10 in >85-year-olds) in the elderly [2]. BRAF mutation is seen in 59% of patients with malignant melanoma [3]. Most of the cases are discovered incidentally during routine skin examination; they typically present at a very early stage [4]. However, about 4% of patients have metastatic disease at presentation [5]. Metastasis occurs through both lymphatics and the hematogenous route simultaneously. As such, it has the potential to metastasize to any organ, and its metastatic behavior is thus unpredictable [6]. Malignant melanoma metastasizes mainly to skin (other areas), lungs (70-87%), liver (54-77%), subcutaneous tissue and lymph nodes (50-75%), heart (40-45%), brain (36-54%), adrenal glands (36-54%), kidneys (35-48%), spleen (30%), GI tract (26-58%), and bone (23%) [6,7]. Although metastasis to bone is well recognized, the incidence is low compared to liver or brain metastasis [8]. However, when it does metastasize, 80%-90% of bone metastases occur to the axial skeleton (skull, ribs, vertebral column, and pelvis) [8]. Malignant melanoma, initially presenting as pathological fracture of the appendicular skeleton, is relatively rare. Our article aims to raise physicians' awareness of this uncommon presentation along with a review of the prognosis and current treatment options.

## Case presentation

**Figure 1:**
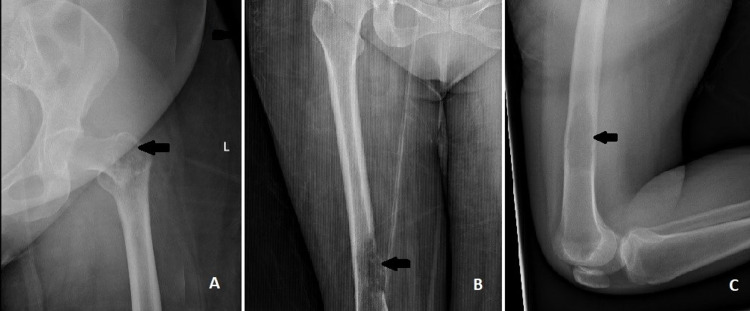
(A) X-ray of the left femur showing intertrochanteric fracture and abnormal, suspicious lesion at the fracture site (arrowhead); (B) and (C) X-ray of the right femur showing osteolytic lesion (arrowhead).

A 45-year-old Caucasian female with no significant past medical history presented to the ED with acute left hip pain that began abruptly when attempting to get out of bed. She heard a pop in her left hip, followed by an inability to bear weight. She denied any falls or trauma. In the ED, initial vital signs and labs were within normal limits. On physical examination, the left lower extremity appeared shortened and externally rotated with decreased range of motion. X-ray demonstrated a displaced intertrochanteric fracture of the left femur and an abnormal, suspicious lesion with a stippled appearance at the fracture site (Figure 1A). A lytic bone lesion was also found in the distal third of the right femur without any fracture (Figure 1B-1C). A pathological fracture due to unknown primary malignancy was suspected due to lytic bone lesions and the trivial nature of her injury.

**Figure 2:**
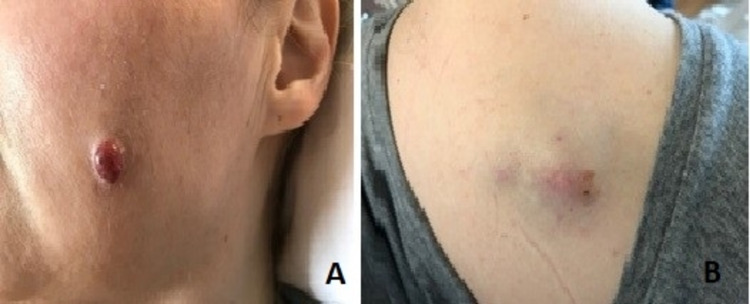
Skin nodules on (A) left cheek (B) right upper back.

Detailed physical examination revealed a skin nodule on the left anterior chest wall measuring about 1 x 1 cm. Similar nodules were also found on the right upper back and left cheek, each measuring approximately 5 x 5 cm and 1 x 1 cm, respectively (Figure 2). Upon further inquiry, the patient reported noticing these lesions that had increased in size over several months prior to presentation. However, she failed to receive any medical care due to her social and family circumstances.

**Figure 3:**
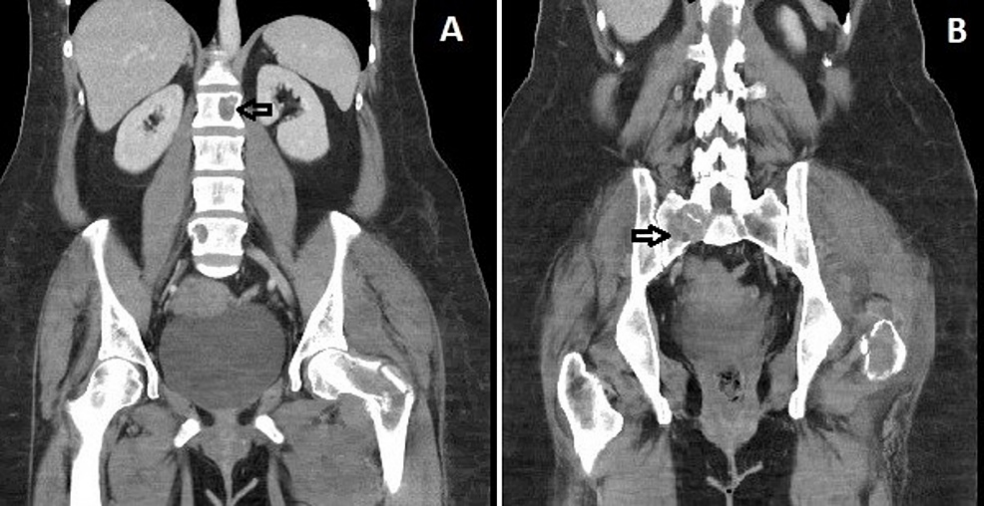
CT scan showing osteolytic bone lesions in (A) lumbar spine (B) ilium.

To better delineate the suspicious femur lesion found on X-ray and to identify the primary malignancy, CT of the chest/abdomen/pelvis (CT C/A/P) was obtained, which showed multiple lytic lesions in the lumbar vertebral bodies, rib cage, ilium, and metaphysis of the left femur (Figure 3). In addition, numerous metastatic soft tissue nodules and lesions in the left adrenal gland, lung, and ovary were also found on CT C/A/P. Based on the history, physical examination, and imaging findings, a broad differential for the site of primary malignancy, including breast cancer, multiple myeloma, ovarian cancer, and melanoma, were considered.

**Figure 4:**
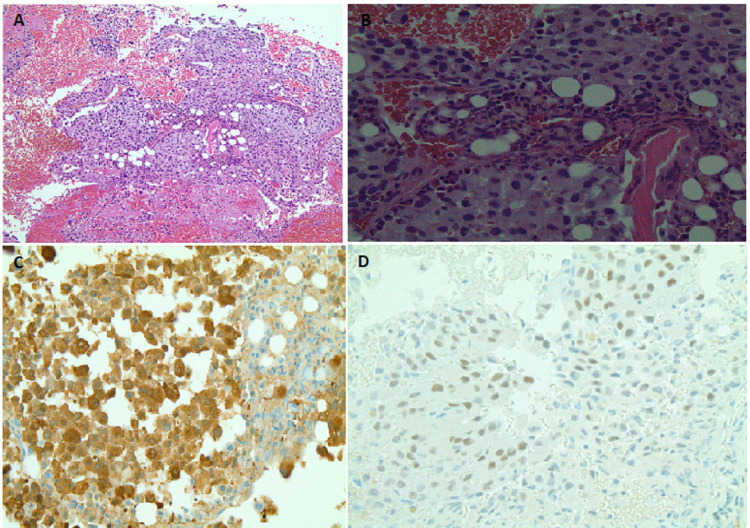
Pathology images from bone biopsy showing metastatic melanoma around normal bone marrow elements; (A) low magnification, (B) high magnification, (C) S100 cytoplasmic staining, (D) SOX 10 nuclear staining.

Additional workup showed a normal pattern on serum protein electrophoresis (SPEP) and urine protein electrophoresis (UPEP) with a mild increase in erythrocyte sedimentation rate (ESR) (37 mm/hr) and C-reactive protein (CRP) (2.0 mg/dL). The patient underwent surgical stabilization of the fracture followed by an open biopsy of the suspicious lesion at the fracture site, which revealed malignant melanoma with BRAF V600E mutation (Figure 4).

CT C/A/P revealed a stage IV (cTX, cN2, cM1b(1)) (American Joint Committee on Cancer [AJCC] cancer staging 8th edition) metastatic melanoma with extensive metastasis to soft tissue, lung, bone, adrenal gland, and mediastinal lymph nodes. MRI brain was, however, negative for any metastatic or malignant process. In addition, her serum lactate dehydrogenase (LDH) was elevated (939 U/L) more than four times the upper limit of normal. Following staging, she was started on palliative radiation therapy followed by targeted therapy, with dabrafenib plus trametinib along with denosumab by the oncology team. Although a favorable response to treatment was noted initially as evidenced by a decrease in size of the soft tissue lesions, a positron emission tomography-computed tomography (PET-CT) scan performed at a three-month follow-up showed a progression of her disease with new metastatic foci in the liver, lungs, and adrenals. Shortly thereafter, the patient's clinical course was complicated by the development of hemoperitoneum due to bleeding from her large hepatic metastasis requiring hospitalization for paracentesis, hepatic embolization, and hepatic wedge resection. Due to the progression of her disease despite targeted therapy, a plan to initiate immunotherapy post clinical stability and recovery was discussed with the patient. However, she developed recurrent hemoperitoneum, GI bleeding, and respiratory failure requiring intubation and mechanical ventilation. Despite prolonged aggressive treatment in the ICU, her clinical condition failed to improve, and she was transitioned to comfort care per her family's wishes. She was deceased shortly after that.

## Discussion

Bone is the third most frequent site of metastasis after lung and liver [9]. Bone metastases are classified as osteolytic, osteoblastic, or mixed based on the primary mechanism of interference with normal bone remodeling [9]. OPG-RANKL-RANK pathway maintains an appropriate balance between the action of osteoblasts and osteoclasts in normal bone remodeling [10]. Bone metastasis causes tumor-induced alterations of this pathway, thereby promoting enhanced osteoclast formation, accelerated bone resorption, and bone loss [10]. Most of the osteolytic bone lesions in adults occur from the metastatic spread of malignant tumors. The most common are multiple myeloma, breast cancer, renal cell carcinoma, non-small cell lung cancer, non-Hodgkin's lymphoma, and thyroid cancer [9]. Patients typically present with localized pain and functional impotence, which may signify a pathological fracture [7]. As seen in our case, approximately 3-4% of patients who present with pathological fractures have unknown primary malignancy at presentation. As such, a thorough history and physical examination, followed by focussed laboratory testing, radiographic staging, and biopsy of the bone lesions, are warranted to confirm the diagnosis. Bone metastasis of malignant melanoma is typically seen as osteolytic lesions of the axial skeleton in patients with advanced disease [7]. Extra-axial skeletal metastasis is relatively rare. In our extensive literature search using PubMed, MEDLINE, and Google Scholar using search terms "metastatic melanoma," "bone metastasis," and "pathological fractures," we found very few cases of melanoma with extra-axial bone metastasis (Table 1) [7, 8, 11, 12]. In these cases, the mean age at presentation was 58 years with a female preponderance. Pain or functional impotence was the chief complaint on presentation, indicating a pathological fracture. The femur and tibia were more commonly involved than the humerus. Most of these cases had a known diagnosis of melanoma on presentation, unlike our patient, who presented with pathological fracture. Further workup to identify the unknown primary malignancy revealed metastatic melanoma. Chemotherapy was the main treatment modality in most of these cases. 

**Table 1:** Clinical and pathological data of patients with melanoma and skeletal metastasis obtained by literature review. NA: Data not available.

S. No	Author [Reference No.]	Age	Sex	Presenting Symptom	Primary site/Histological type	Metastatic site	Treatment	Overall Survival (Months)
1	Costache M et al. [7]	44	F	Functional impotence	NA/Malignant melanoma	Femur and humerus	Chemotherapy	NA
2	Gómez-León N et al. [8]	61	F	Mild local pain	NA/Superficial spreading melanoma	Trochanter of the femur	Chemotherapy	37
3	Gómez-León N et al. [8]	38	F	Mild local pain	NA/Malignant melanoma	Knee	BRAF inhibitors	24
4	Herrera-Perez M et al. [11]	39	M	Pain	Abdominal flank/ Malignant melanoma	Distal tibia	Chemotherapy + local radiotherapy	NA
5	Huang KY et al. [12]	67	F	NA	Heel/Acral lentiginous melanoma	Femur	Radiotherapy	38.97
6	Huang KY et al. [12]	70	F	NA	Leg/Malignant melanoma	Femur	Chemotherapy	13.67
7	Huang KY et al. [12]	67	M	NA	Foot/Acral lentiginous melanoma	Tibia	Chemotherapy	7.77
8	Huang KY et al. [12]	74	F	NA	Leg/Malignant melanoma	Tibia	Chemotherapy	10.76

Based on the AJCC melanoma database (7th edition), one-year survival rates among patients with stage IV melanoma were found to be 62% for M1a (distant metastasis to the skin, soft tissue including muscle, and/or non-regional lymph node), 53% for M1b (distant metastasis to lung), and 33% for M1c (all other visceral metastases and/or any distant metastasis). As such, patients with non-pulmonary visceral metastasis were found to have a poor prognosis compared to patients with soft tissue (including skin and lymph nodes) or lung metastasis without other visceral involvement [13]. Melanoma-specific survival analysis of patients with stage IV was not performed for the eighth edition AJCC staging system [14]. A recent retrospective survey on bone metastasis in melanoma patients by Mannavola F et al. revealed a median survival from the onset of bone metastasis to be 10.7 months [3]. Multivariate analysis revealed the number of bone metastases, elevated serum LDH, poor performance status, three or more metastatic sites, and high tumor burden as negative prognostic factors [3]. No difference in overall survival (OS) has been observed between metastasis of melanoma to appendicular skeleton compared to axial skeleton [3, 7]. Distant visceral metastases and an elevated serum LDH level explain the poor prognosis of our patient in the present case. 

Over the last decade, the treatment landscape has changed and continues to evolve, leading to rapidly improving prognoses. Recently, BRAF/MEK targeted therapy and immunotherapy with anti-CTLA-4 and anti-PD-1/PDL-1 have shown improved response rates and OS. Pooled analyses of two phase III clinical trials, COMBI-v and COMBI-d showed five-year progression-free survival (PFS) and OS rates of 19% and 34%, respectively, in patients with unresectable metastatic melanoma treated with dabrafenib and trametinib [15]. CheckMate 067 evaluated the role of antiCTLA-4 (ipilimumab) and anti-PD-1 (nivolumab) combination therapy in treatment-naïve advanced melanoma patients. The combination therapy resulted in an overall response rate of 58% and a five-year OS rate of 60% in BRAF-mutated melanoma [16]. However, the choice of first-line therapy is a complicated decision. It is currently unknown whether BRAF/MEK targeted therapy or immunotherapy alone or the combination of targeted therapy with immunotherapy should be considered the first line of therapy in BRAF-mutated melanoma [16]. KEYNOTE-022, a phase II clinical trial involving metastatic or unresectable melanoma patients, evaluated the combination of dabrafenib and trametinib with pembrolizumab. The triple therapy showed a PFS of 41% at 24 months compared to 16% for BRAF/MEK therapy. Median OS was not reached for triple therapy and was 26.3 months for BRAF/MEK therapy [16]. Another clinical trial, IMspire150 evaluated atezolizumab (anti-PDL-1) with cobimetinib and vemurafenib (BRAF/MEK inhibitors) (triple therapy) on advanced, locally resectable melanoma patients. The results showed a PFS of 15.1 months with triple therapy compared to 10.6 months with BRAF/MEK therapy. The PFS rate was 54% at 12 months, with an overall response rate of 66% (median duration of response of 21 months) and a median OS of 28.8 months with triple therapy [16, 17]. 

For patients with bone metastasis, bone-targeting agents (BTAs), such as bisphosphonate (zoledronic acid) and RANK-L inhibitors (denosumab), are found to improve bone pain and reduce the development of skeletal-related events (SREs) by restoring the physiological bone turnover [3]. Some studies have shown a synergistic effect of RANK-L inhibitors when combined with immunotherapy in patients with bone metastasis from malignant melanoma, suggesting a potential clinical benefit [3]. In our literature review, we found one case that has reported regression of bone metastasis in a patient with malignant melanoma following treatment with localized radiotherapy combined with systemic zoledronate [18]. Review of results from three randomized controlled trials (MDX010-20, KEYNOTE-002, and CheckMate 067) suggested that ipilimumab, nivolumab, and pembrolizumab, as monotherapy or combination therapy of nivolumab plus ipilimumab or ipilimumab plus gp100 vaccine, either improved or maintained the health-related quality of life (HRQOL) in advanced melanoma patients [19].

Our patient had disease progression on BRAF/MEK targeted therapy, and initiation of immunotherapy was considered. Unfortunately, the patient was hospitalized due to the development of life-threatening complications from her metastatic disease before initiation of immunotherapy. Currently, cellular therapy using tumor-infiltrating lymphocytes (TILs) is under trial in patients with metastatic melanoma who had progressive disease on targeted therapy and immunotherapy [20]. Sarnaik AA et al. reported the results of the phase II trial and showed promising results with objective response achieved in 36% of patients and a disease control rate of 81% [20]. There are currently 12 actively recruiting phase III/IV clinical trials on stage IV melanoma patients exploring several new treatment avenues, including but not limited to the combination of granulocyte-macrophage colony-stimulating factor (GM-CSF) with immunotherapy [NCT02339571], CpGA DNA Toll-like receptor agonist with pembrolizumab [NCT04695977], an immunomodulatory vaccine against indoleamine 2,3-dioxygenase (IDO) with nivolumab [NCT05155254], and HDAC inhibitor with nivolumab [NCT04674683]. More clinical trials are required to explore the treatment options for patients who have progression on targeted therapy and immunotherapy. 

## Conclusions

In conclusion, we described the case of a patient who failed to seek medical attention for ongoing skin lesions and presented with widespread metastatic disease. Though bone metastasis is seen with melanoma, extra-axial skeletal metastasis is relatively a rare phenomenon. Therefore, a high index of suspicion is needed in patients who present with an appendicular fracture without trauma. Our case highlights the aggressive nature of untreated melanoma. Targeted therapy with BRAF/MEK inhibitors and immunotherapy with immune checkpoint inhibitors showed better outcomes in terms of OS and PFS. Our case also highlights the need for further clinical trials to explore treatment options for patients who have disease progression on targeted therapy and immunotherapy. 
